# Estrogen receptor β regulates sex‐dependent airway mechanics and inflammation in a murine model of allergen exposure

**DOI:** 10.14814/phy2.70948

**Published:** 2026-05-31

**Authors:** Carolyn Damilola Ekpruke, Dustin Rousselle, Maksat Babayev, Omar Borges‐Sosa, Rachel Alford, Lyidia Dinwiddie, David Michael Merritt, Christopher Michael Hemmerich, Douglas B. Rusch, Sarah Bradley, Matthew Louis Retzner, Erik Parker, Patricia Silveyra

**Affiliations:** ^1^ Department of Environmental and Occupational Health School of Public Health, Indiana University Bloomington Indiana USA; ^2^ Center for Genomics and Bioinformatics, Indiana University Bloomington Indiana USA; ^3^ Biostatistics Consulting Center, Department of Epidemiology and Biostatistics, School of Public Health Indiana University Bloomington Indiana USA; ^4^ School of Medicine Indiana University Indianapolis Indiana USA

**Keywords:** allergic airway inflammation, asthma, estrogen receptor beta (ERβ), house dust mite (HDM), sex differences

## Abstract

Sex differences in asthma severity have been reported; however, the specific contribution of estrogen receptor β (ERβ) remains incompletely defined. We tested the hypothesis that ERβ modulates sex‐specific physiological responses to chronic allergen exposure using C57BL/6J wild‐type (WT) and ERβ‐deficient (Esr2^−^/^−^) male and female mice subjected to 5 weeks of house dust mite (HDM) challenge. Lung mechanics were assessed using flexiVent and integrated with histopathology and whole‐lung transcriptomics. We found that HDM exposure increased total respiratory resistance (Rrs), Newtonian resistance (Rn), and tissue elastance (H), with the greatest impairment in female Esr2^−^/^−^ mice, significantly exceeding WT females. These changes were accompanied by marked peribronchial inflammation and mucus metaplasia, particularly in females. ERβ deficiency also produced compartment‐specific effects. Transcriptomic analyses revealed disruption of epithelial differentiation, immunoglobulin gene expression, and metabolic pathways, with downregulation of humoral and epithelial programs in Esr2^−^/^−^ females. Collectively, these findings identify ERβ as a critical determinant of sex‐specific airway mechanics and inflammatory remodeling during allergen exposure. We conclude that ERβ protects the female lung from exaggerated functional decline, mucus metaplasia, and tissue stiffening, while exerting distinct spatial and immunological effects in males, highlighting ERβ signaling as a target for sex‐informed asthma therapeutics.

## 1 INTRODUCTION

Asthma is a chronic inflammatory disease of the airways characterized by reversible airflow obstruction, airway hyperresponsiveness, and structural remodeling. Affecting over 300 million individuals globally, asthma continues to impose a major health and economic burden despite the availability of effective treatments (Cao et al., 2025). The disease is distinguished by its marked sex‐based disparity in prevalence and severity: before puberty, asthma is more common in males, but after puberty, adult females exhibit higher incidence, more frequent exacerbations, and more severe disease (Ekpruke & Silveyra, 2022; Pinkerton et al., 2015; Silveyra et al., 2026). This shift coinciding with hormonal maturation strongly implicates sex hormones and their receptors as central modulators of airway inflammation and remodeling (Yung et al., 2018).

Among these hormonal pathways, estrogen signaling plays a particularly complex and context‐dependent role in lung immunity and physiology. Estrogens act through multiple receptors, including the classical nuclear receptors estrogen receptor α (ERα) and estrogen receptor β (ERβ), as well as the membrane‐associated G‐protein–coupled estrogen receptor (GPER), to regulate gene expression and cell signaling in a wide range of tissues (Arterburn & Prossnitz, 2023; Chen et al., 2022; Hou & Adzika, 2025; Prossnitz & Barton, 2011). Both ERα and ERβ act as ligand‐activated transcription factors that bind estrogen response elements (EREs) in genomic DNA and also exert rapid nongenomic signaling through kinase cascades such as MAPK, PI3K/AKT, and ERK1/2 (Kovats, 2015). Although ERα is frequently associated with pro‐inflammatory and proliferative signaling, ERβ has emerged as a critical mediator of anti‐inflammatory and tissue‐protective functions (Gong et al., 2014; Song et al., 2022).

ERβ is expressed throughout the respiratory tract, including in airway epithelial cells, fibroblasts, airway smooth muscle, endothelial cells, macrophages, and lymphocytes, indicating its potential as a global regulator of pulmonary homeostasis (Ekpruke & Silveyra, 2022; Silveyra et al., 2026). Through genomic and nongenomic mechanisms, ERβ modulates inflammatory gene transcription, oxidative stress, mitochondrial function, and cell differentiation. In immune cells, ERβ restrains the activation of pro‐inflammatory transcription factors such as NF‐κB and AP‐1 and promotes resolution pathways that attenuate cytokine production and leukocyte recruitment (Keselman et al., 2017). In macrophages, ERβ activation drives polarization toward an anti‐inflammatory M2 phenotype, thereby limiting tissue damage and promoting repair (Keselman et al., 2017; Son & Im, 2024). In airway smooth muscle, ERβ signaling influences actin cytoskeletal dynamics and calcium homeostasis, controlling cell contractility and migration (Ambhore et al., 2020, 2024). Collectively, these actions highlight ERβ as a pleiotropic regulator of immune–structural interactions in the lung.

Beyond immunoregulation, ERβ also governs epithelial barrier integrity and tissue remodeling, processes central to asthma pathophysiology. Airway epithelial cells constitute the first line of defense against inhaled allergens and pollutants, and their dysfunction amplifies airway inflammation. ERβ signaling supports epithelial repair by maintaining tight‐junction integrity, limiting epithelial‐mesenchymal transition (EMT), and promoting antioxidant responses through mitochondrial biogenesis and redox control (Chen, Russo, et al., 2007; Pedram et al., 2010; Rius‐Pérez et al., 2020). These functions are critical for preventing chronic airway injury and fibrotic remodeling. ERβ activation also suppresses the production of matrix metalloproteinases (MMPs) and profibrotic cytokines, thereby modulating extracellular matrix turnover and limiting structural remodeling (Pedram et al., 2010). Furthermore, ERβ's influence on mitochondrial gene expression and energy metabolism links hormonal signaling to metabolic resilience, an emerging determinant of chronic airway inflammation and corticosteroid resistance (Novelle et al., 2023; Rius‐Pérez et al., 2020).

Despite accumulating evidence supporting ERβ's protective role in inflammatory and fibrotic processes, its precise mechanisms of action in allergic airway inflammation remain incompletely defined. Prior studies have largely focused on ERα or failed to distinguish between receptor subtypes, leaving the specific contribution of ERβ unclear. Moreover, how ERβ signaling integrates immune and epithelial responses during chronic allergen exposure, and whether these effects differ between sexes, remains unknown. Given the well‐established female bias in asthma prevalence and severity, understanding the sex‐specific roles of ERβ in airway inflammation is essential to unraveling the biological basis of this disparity (Ekpruke & Silveyra, 2022; Silveyra et al., 2026).

In this study, we used a chronic house dust mite (HDM) model of allergic airway inflammation in ERβ knockout (Esr2^−^/^−^) and wild‐type (WT) mice of both sexes to define the molecular, cellular, and physiological consequences of ERβ loss. The HDM model reproduces many hallmarks of human asthma, including eosinophilic infiltration, mucus hypersecretion, epithelial barrier dysfunction, and airway remodeling. By integrating lung mechanics, histopathological assessment, and whole‐lung transcriptomic analysis, we aimed to dissect the ERβ‐dependent pathways that govern airway inflammation and remodeling. We hypothesized that ERβ acts as a central regulator of lung homeostasis, maintaining epithelial integrity, modulating immune activation, and preserving tissue compliance during chronic allergen exposure, with these effects exhibiting sexual dimorphism. Our findings demonstrate that the loss of ERβ amplifies allergen‐induced inflammation, disrupts humoral and epithelial gene networks, and exacerbates airway hyperresponsiveness in a sex‐dependent manner. Together, these results position ERβ as a key molecular nexus linking estrogen signaling to airway inflammation, epithelial injury, and remodeling, providing new mechanistic insight into sex‐biased asthma pathogenesis and potential avenues for hormone‐targeted therapy.

## 2 MATERIALS AND METHODS

### 2.1 Animals

Male and female Esr2^−^/^−^ (ERβ knockout) and WT littermate mice on a C57BL/6J background were used at 8–10 weeks. Mice were bred from pairs obtained from The Jackson Laboratory (Strain #:004745; B6.129P2‐Esr2^tm1Unc^/J) and housed under specific pathogen–free conditions in the Indiana University Bloomington Animal facility vivarium, with ad libitum access to food and water and a 12 h light/dark cycle. Mice were maintained on standard rodent chow (LabDiet 5001 Laboratory Rodent Diet, PMI Nutrition International, St. Louis, MO, USA). All animal procedures were approved by the Bloomington Institutional Animal Care and Use Committee (BIACUC protocols # 24‐020; 21‐012) and conducted in accordance with NIH guidelines for the care and use of laboratory animals. Genotypes were confirmed by PCR analysis using ear punch tissue collected at 3–4 weeks of age following The Jackson Laboratory's protocol. Mice aged 8–10 weeks were grouped by genotype and sex for experiments. Group sizes varied by experiment (*n* = 3–10) and are specified in figure legends.

### 2.2 House dust mite (HDM) exposure

Chronic allergic airway inflammation was induced using house dust mite (HDM) extract *Dermatophagoides pteronyssinus* and *Dermatophagoides farinae* (Citeq Biologics, Groningen, Netherlands; Cat. No. 02.50.85.10). Mice received intranasal instillations of 25 μg HDM in 50 μL PBS or 50 μL sterile PBS (control) five times per week for five consecutive weeks under light isoflurane anesthesia. Mice were euthanized 72 h after the final challenge for assessment of lung mechanics, histology, and transcriptomics.

### 2.3 Lung mechanics

Airway physiology was evaluated using the flexiVent FX system (SCIREQ, Montreal, Canada) to measure total respiratory system resistance (Rrs) and elastance (Ers), which represent the overall resistive and elastic properties of the respiratory system. Newtonian resistance (Rn) reflects conducting (central) airway caliber and bronchoconstriction, whereas tissue damping (G) and tissue elastance (H) reflect peripheral tissue mechanics, including energy dissipation, parenchymal stiffness, and heterogeneity of lung impedance. Mice were anesthetized with ketamine/xylazine 100/10 mg/kg intraperitoneally, tracheostomized with an 18‐gage cannula, and mechanically ventilated (tidal volume 10 mL/kg, 150 breaths/min, PEEP 3 cmH_2_O). To suppress spontaneous breathing, pancuronium bromide (10 mg/kg) was administered intraperitoneally. Aerosolized methacholine (Sigma‐Aldrich, Cat. No. 1396364) was administered in increasing doses (0, 3.13, 12.5, 25, 50, and 100 mg/mL) using the integrated nebulizer. Airway resistance parameters were measured after each dose, and data were analyzed using the single‐compartment and constant‐phase models in flexiWare software (version 8.2, SCIREQ).

### 2.4 Histology and morphometric analysis

Mice were anesthetized (ketamine/xylazine, 100/10 mg/kg), exsanguinated, and perfused via the trachea with 5 mL of 10% neutral buffered formalin delivered by gravity (25 cm height). Lungs were fixed overnight in formalin and transferred to 70% ethanol for paraffin embedding. Lung sections (4 μm thick) were stained with hematoxylin and eosin (H&E) for evaluation of inflammation and tissue morphology, and Alcian Blue/Periodic Acid–Schiff (AB‐PAS) for detection of mucus‐producing goblet cells. Staining was performed by the Indiana University School of Medicine Histology Core. Perivascular and peribronchial inflammatory cell infiltration was quantified on H&E‐stained sections using QuPath (v. 0.4.3) software and expressed as cells/mm^2^ of airway or vessel area. Goblet cell hyperplasia was determined by counting the number of AB‐PAS^+^ epithelial cells per mm^2^ of airway epithelium. At least 5–10 randomly selected airways were analyzed per animal. All analyses were performed by observers blinded to genotype and exposure group.

### 2.5 RNA isolation, library preparation, and sequencing

Total RNA was extracted from snap‐frozen whole lungs using the Zymo Direct‐zol RNA Miniprep Kit (Zymo Research, Irvine, CA; Cat. No. R2050) following the manufacturer's protocol, including in‐column DNase I treatment to remove genomic DNA. RNA purity and concentration were determined using a NanoDrop spectrophotometer (Thermo Fisher Scientific), and RNA integrity was assessed with an Agilent 2100 Bioanalyzer. Samples with an RNA integrity number (RIN) ≥7.0 were used for sequencing.

RNA‐seq libraries were prepared using the NEBNext Ultra II Directional RNA Library Prep Kit for Illumina (New England Biolabs, Ipswich, MA; Cat. No. E7760) with poly(A) + selection. Library quality was confirmed on an Agilent Bioanalyzer, and indexed libraries were pooled equimolarly. Sequencing was performed at the Indiana University Center for Genomics and Bioinformatics (CGB) on an Illumina NovaSeq 6000 platform using paired‐end 150‐bp reads. Libraries were sequenced across two independent NovaSeq runs (CGB Project ID GSF3966), generating a total of approximately 2.56 billion pass‐filter reads (~30–40 million reads per sample).

### 2.6 Read processing and differential expression analysis

Demultiplexed FASTQ files were assessed for quality using FastQC (v0.11.9) and trimmed for adapters and low‐quality bases using Trimmomatic (v0.39). Clean reads were aligned to the mouse reference genome (GRCm38/mm10) using STAR aligner (v2.7), and gene‐level counts were quantified using featureCounts (Subread v2.0). Differential expression analysis was conducted in R (v4.3.0) using DESeq2 (v1.38.0), using a factorial design. Genes with an adjusted *p*‐value (FDR <0.05) and |log_2_ fold change| ≥1 were considered differentially expressed. Volcano plots were generated using base R packages to visualize sample clustering and group differences.

### 2.7 Pathway and network analysis

Differentially expressed gene (DEG) lists were analyzed using Ingenuity Pathway Analysis (IPA) (Qiagen) to identify enriched canonical pathways, upstream regulators, and molecular interaction networks. Canonical pathways were considered significant at Benjamini‐Hochberg FDR <0.05; activation states were inferred using IPA *Z*‐scores.

### 2.8 Statistical analyses

All statistical analyses were performed using R (version ≥4.2.0). Data are presented as estimated marginal means (emmean) ± standard error (SE) unless otherwise stated. Statistical significance was defined as *p* < 0.05.

#### 2.8.1 Lung mechanics

FlexiVent‐derived lung mechanics parameters were analyzed using linear mixed‐effects models to account for repeated measurements within animals across methacholine doses. For each outcome, a single model was fitted that included genotype (WT vs. Esr2^−^/^−^), exposure (PBS vs. HDM), sex (males vs. females), and methacholine dose as fixed effects, along with all lower‐ and higher‐order interaction terms up to the four‐way interaction. Mouse identity was included as a random intercept to account for within‐subject correlation. Degrees of freedom were estimated using the Kenward–Roger approximation, which provides robust inference for unbalanced designs. Global significance of fixed effects and interactions was assessed using Type III ANOVA. When significant main effects or interactions were detected, post hoc pairwise comparisons were performed using estimated marginal means with Tukey's adjustment for multiple comparisons. Where relevant, analyses were stratified by sex, genotype, or exposure to facilitate biologically interpretable contrasts.

#### 2.8.2 Histopathology

Quantitative analyses of perivascular inflammation, peribronchial inflammation, and PAS^+^ goblet‐cell density were performed using linear models including genotype, exposure, sex, and their interactions as fixed effects. Cell density measurements (cells/mm^2^) were derived from multiple nonoverlapping airway or vascular regions per animal and averaged to yield a single value per mouse. Global effects were assessed using Type III ANOVA, followed by Tukey‐adjusted post hoc comparisons where appropriate.

#### 2.8.3 Transcriptomics

RNA‐sequencing data were analyzed using established pipelines. Differential gene expression was assessed using generalized linear models with genotype, sex, and exposure as factors. Genes were considered differentially expressed based on an adjusted *p*‐value threshold (false discovery rate, FDR) as specified in the RNA‐seq Methods section. Principal component analysis and pathway enrichment analyses were used for exploratory and mechanistic interpretation.

#### 2.8.4 Data integrity and reproducibility

Sample sizes for each experiment are indicated in the figure legends. No data points were excluded unless justified by predefined technical criteria. Investigators were blinded during histological scoring and image quantification. All statistical tests were two‐sided.

## 3 RESULTS

### 3.1 Estrogen receptor β protects against allergen‐induced airway mechanical dysfunction in a sex‐dependent manner

To define the physiological consequences of ERβ deficiency during chronic allergen exposure, we quantified airway and tissue mechanics across increasing methacholine doses in all groups.

#### 3.1.1 Total respiratory resistance (Rrs)

**FIGURE 1:**
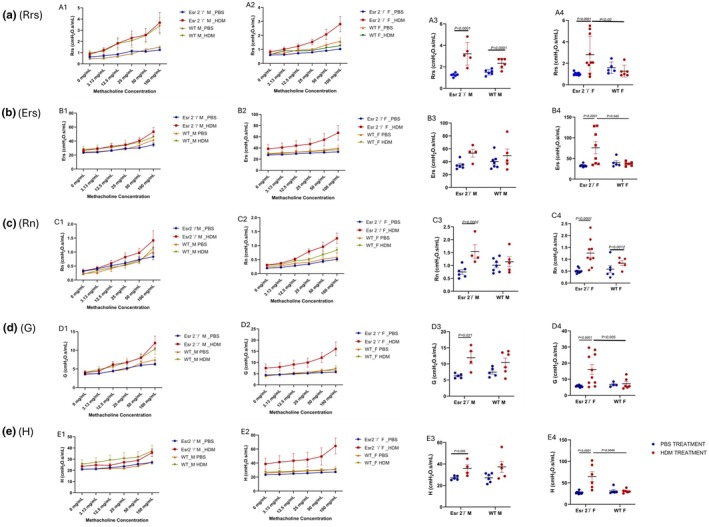
ERβ regulates airway mechanical responses following chronic HDM exposure in a sex‐dependent manner. (A1, A2) Methacholine dose–response curves for total respiratory resistance (Rrs) in male and female WT and Esr2^−^/^−^ mice following chronic PBS or HDM exposure. (A3, A4) Quantification of Rrs at 100 mg/mL methacholine demonstrating exaggerated airway resistance in HDM‐treated Esr2^−^/^−^ mice, particularly females. (B1, B2) Respiratory system elastance (Ers) dose–response curves in male and female mice. (B3, B4) Endpoint Ers measurements at 100 mg/mL methacholine showing increased lung stiffness in HDM‐treated Esr2^−^/^−^ females relative to WT controls. (C1, C2) Newtonian resistance (Rn) dose–response curves demonstrating enhanced conducting airway narrowing following HDM exposure. (C3, C4) Quantification of Rn at 100 mg/mL methacholine indicating increased central airway resistance in Esr2^−^/^−^ mice. (D1, D2) Tissue damping (G) dose–response curves reflecting peripheral tissue resistance and energy dissipation. (D3, D4) Endpoint measurements of G demonstrating increased small‐airway and parenchymal resistance in HDM‐treated Esr2^−^/^−^ mice, particularly females. (E1, E2) Tissue elastance (H) dose–response curves reflecting parenchymal stiffness and tissue heterogeneity. (E3, E4) Quantification of tissue elastance at 100 mg/mL methacholine showing severe distal lung stiffening in HDM‐treated Esr2^−^/^−^ females. Data are presented as mean ± SEM, *n* = 4–10/group. Exact *p*‐values are indicated on the graphs. Blue symbols represent PBS‐treated mice and red symbols represent HDM‐treated mice. WT, wild type; HDM, house dust mite.

Linear mixed‐effects modeling revealed no significant four‐way interaction, but significant effects of exposure (*p* < 0.001), methacholine dose (*p* < 0.001), exposure × methacholine dose (*p* < 0.001), sex × methacholine dose (*p* = 0.002) and genotype × exposure × methacholine dose (*p* = 0.04), indicating that HDM‐induced increases in airway resistance are methacholine dose dependent and modified by sex and genotype. At low methacholine concentrations (0–3.13 mg/mL) HDM did not significantly change Rrs in any group, although numerical increases were apparent (Figure 1A1–A4). The earliest significant exposure effect was observed at 12.5 mg/mL in WT males (HDM 1.81 ± 0.23 cmH_2_O·s/mL vs. PBS 0.66 ± 0.22 cmH_2_O·s/mL; *p* = 0.0088); WT females remained unchanged. At 25 mg/mL HDM significantly elevated Rrs in Esr2^−^/^−^ females (1.69 ± 0.20 vs. 0.80 ± 0.22 cmH_2_O·s/mL; *p* = 0.0195), Esr2^−^/^−^ males (2.29 ± 0.23 vs. 1.04 ± 0.22 cmH_2_O·s/mL; *p* = 0.0089) and WT males (2.06 ± 0.23 vs. 0.91 ± 0.22 cmH_2_O·s/mL; *p* = 0.0085), while WT females again showed no effect. The pattern intensified at 50 mg/mL, with marked increases in Esr2^−^/^−^ females (2.30 ± 0.20 vs. 0.91 ± 0.22 cmH_2_O·s/mL; *p* = 0.0004), Esr2^−^/^−^ males (2.60 ± 0.23 vs. 1.15 ± 0.22 cmH_2_O·s/mL; *p* = 0.0026) and WT males (2.53 ± 0.23 vs. 1.26 ± 0.22 cmH_2_O·s/mL; *p* = 0.0040); WT females were unaffected. At the maximal methacholine dose (100 mg/mL) HDM produced the largest increases: Esr2^−^/^−^ females 3.04 ± 0.35 versus PBS 1.03 ± 0.43 cmH_2_O·s/mL (*p* < 0.0001), Esr2^−^/^−^ males 3.69 ± 0.54 versus PBS 1.31 ± 0.49 cmH_2_O·s/mL (*p* < 0.0001), and WT males 3.39 ± 0.46 versus PBS 1.50 ± 0.49 cmH_2_O·s/mL (*p* < 0.0001); WT females again remained resistant (HDM 1.43 ± 0.43 vs. PBS 1.59 ± 0.54 cmH_2_O·s/mL; *p* = 0.730). Among HDM‐treated mice at 100 mg/mL, Esr2^−^/^−^ females had significantly higher Rrs than WT females (3.04 ± 0.35 vs. 1.43 ± 0.43 cmH_2_O·s/mL; *p* = 0.0267); WT females had significantly lower Rrs than Esr2^−^/^−^ males (*p* = 0.0047) and WT males (*p* = 0.0080), whereas Esr2^−^/^−^ and WT males did not differ (*p* = 0.964).

#### 3.1.2 Respiratory system elastance (Ers)

Methacholine dose was a dominant determinant of Ers (*p* < 0.001), with significant exposure × dose (*p* = 0.001) and genotype × exposure × dose (*p* = 0.002) interactions. HDM produced pronounced increases in Ers in female Esr2^−^/^−^ mice: at 25 mg/mL the HDM‐PBS contrast was 16.12 ± 7.32 cmH_2_O·s/mL (*p* = 0.0316), at 50 mg/mL it was 22.68 ± 7.32 cmH_2_O·s/mL (*p* = 0.0030) and at 100 mg/mL it was 34.09 ± 7.32 cmH_2_O·s/mL (*p* < 0.0001) (Figure 1B1–B4). A smaller but significant HDM effect was detected in Esr2^−^/^−^ males at 12.5 mg/mL (0.9593 ± 0.374 cmH_2_O·s/mL; *p* = 0.0428); however, WT mice showed no significant HDM–PBS differences at corresponding methacholine doses. Genotype comparisons at 100 mg/mL (Figure 1B3, B4) among HDM‐treated group confirmed an ERβ‐dependent effect as HDM‐treated Esr2^−^/^−^ females had substantially higher Ers than HDM‐treated WT females (67.1 ± 6.58 vs. 37.1 ± 8.49 cmH_2_O·s/mL; *p* = 0.045), and Esr2^−^/^−^ females nearly doubled Ers relative to PBS controls (67.1 ± 6.58 vs. 33.0 ± 7.36 cmH_2_O·s/mL; *p* < 0.0001).

#### 3.1.3 Newtonian resistance (Rn)

There were significant main effects of exposure (*p* < 0.001), sex (*p* = 0.016) and methacholine dose (*p* < 0.001) and significant exposure × dose (*p* < 0.001), sex × dose (*p* < 0.001) and genotype × exposure × dose (*p* = 0.026) interactions, reflecting methacholine dose‐dependent effects of HDM on central airway resistance that were modified by sex and genotype. In Esr2^−^/^−^ females, HDM significantly increased Rn at 12.5 mg/mL (HDM 0.507 ± 0.073 vs. PBS 0.267 ± 0.082 cmH_2_O·s/mL; *p* = 0.0319), 25 mg/mL (0.694 ± 0.073 vs. 0.327 ± 0.082 cmH_2_O·s/mL; *p* = 0.0012), 50 mg/mL (0.992 ± 0.073 vs. 0.408 ± 0.082 cmH_2_O·s/mL; *p* < 0.0001) and 100 mg/mL (1.205 ± 0.073 vs. 0.488 ± 0.082 cmH_2_O·s/mL; *p* < 0.0001) (Figure 1C1–C4). WT females exhibited significant HDM‐induced increases in Rn at 50 mg/mL (0.757 ± 0.095 vs. 0.387 ± 0.104 cmH_2_O·s/mL; *p* = 0.0097) and 100 mg/mL (0.914 ± 0.095 vs. 0.444 ± 0.104 cmH_2_O·s/mL; *p* = 0.0012) compared to the PBS‐treated group. In males, Esr2^−^/^−^ mice showed an HDM‐PBS difference only at 100 mg/mL (1.415 ± 0.116 vs. 0.833 ± 0.095 cmH_2_O·s/mL; *p* = 0.0002). In contrast, WT males did not exhibit significant contrasts at individual methacholine doses. Notably, although WT females exhibited significant increases in Rn at high methacholine doses, these changes were not accompanied by alterations in global respiratory mechanics or tissue‐level parameters, indicating preserved distal lung function.

#### 3.1.4 Airway tissue damping (G)

HDM exposure (*p* = 0.026) and methacholine dose (*p* < 0.001) significantly affected G, with a genotype × exposure × dose interaction (*p* = 0.019). HDM markedly increased G in Esr2^−^/^−^ females at 3.13 mg/mL (8.00 ± 1.13 vs. 4.59 ± 1.26 cmH_2_O·s/mL; *p* = 0.0484), 12.5 mg/mL (9.12 ± 1.13 vs. 4.92 ± 1.26 cmH_2_O·s/mL; *p* = 0.0154), 25 mg/mL (10.00 ± 1.13 vs. 5.21 ± 1.26 cmH_2_O·s/mL; *p* = 0.0061), 50 mg/mL (12.16 ± 1.13 vs. 5.47 ± 1.26 cmH_2_O·s/mL; *p* = 0.0002) and 100 mg/mL (16.07 ± 1.13 vs. 5.74 ± 1.26 cmH_2_O·s/mL; *p* < 0.0001). Esr2^−^/^−^ males showed a weaker but significant HDM effect at 100 mg/mL (11.95 ± 1.78 vs. 6.29 ± 1.59 cmH_2_O·s/mL; *p* = 0.0211) (Figure 1D1–D4). WT mice did not display significant HDM responses at comparable methacholine doses. At the maximal methacholine dose HDM‐treated Esr2^−^/^−^ females had substantially higher G than HDM‐treated WT females (16.07 ± 1.13 vs. 7.25 ± 1.45 cmH_2_O·s/mL; *p* = 0.005).

#### 3.1.5 Tissue elastance (H)

The full model identified a significant four‐way interaction (genotype × exposure × sex × methacholine dose; *p* = 0.015) together with significant effects of exposure (*p* = 0.005), sex (*p* = 0.039), methacholine dose (*p* < 0.001), genotype: dose (*p* = 0.024), genotype:exposure:sex (*p* = 0.034) and genotype:exposure:dose (*p* = 0.002). Post‐hoc comparisons show that HDM strongly elevated H in females at nearly every methacholine dose (HDM − PBS: 0 mg/mL = 8.71 ± 4.06 cmH_2_O·s/mL, *p* = 0.0369; 3.13 mg/mL = 9.68 ± 4.06, *p* = 0.0210; 12.5 mg/mL = 9.90 ± 4.06, *p* = 0.0185; 25 mg/mL = 10.20 ± 4.06, *p* = 0.0153; 50 mg/mL = 12.28 ± 4.06, *p* = 0.0039; 100 mg/mL = 18.67 ± 4.06, *p* < 0.0001). In males, HDM increased H only at the highest methacholine dose compared to the PBS‐group (100 mg/mL: 9.43 ± 4.62; *p* = 0.005). At 100 mg/mL HDM‐treated Esr2^−^/^−^ females exhibited dramatically higher H than HDM‐treated WT females (64.5 ± 4.67 vs. 30.9 ± 4.67 cmH_2_O·s/mL; *p* = 0.0046), whereas genotype differences were not significant in males (Figure 1E1–E4).

### 3.2 Estrogen receptor β affects allergen‐induced airway inflammation and remodeling in a sex‐dependent manner

**FIGURE 2:**
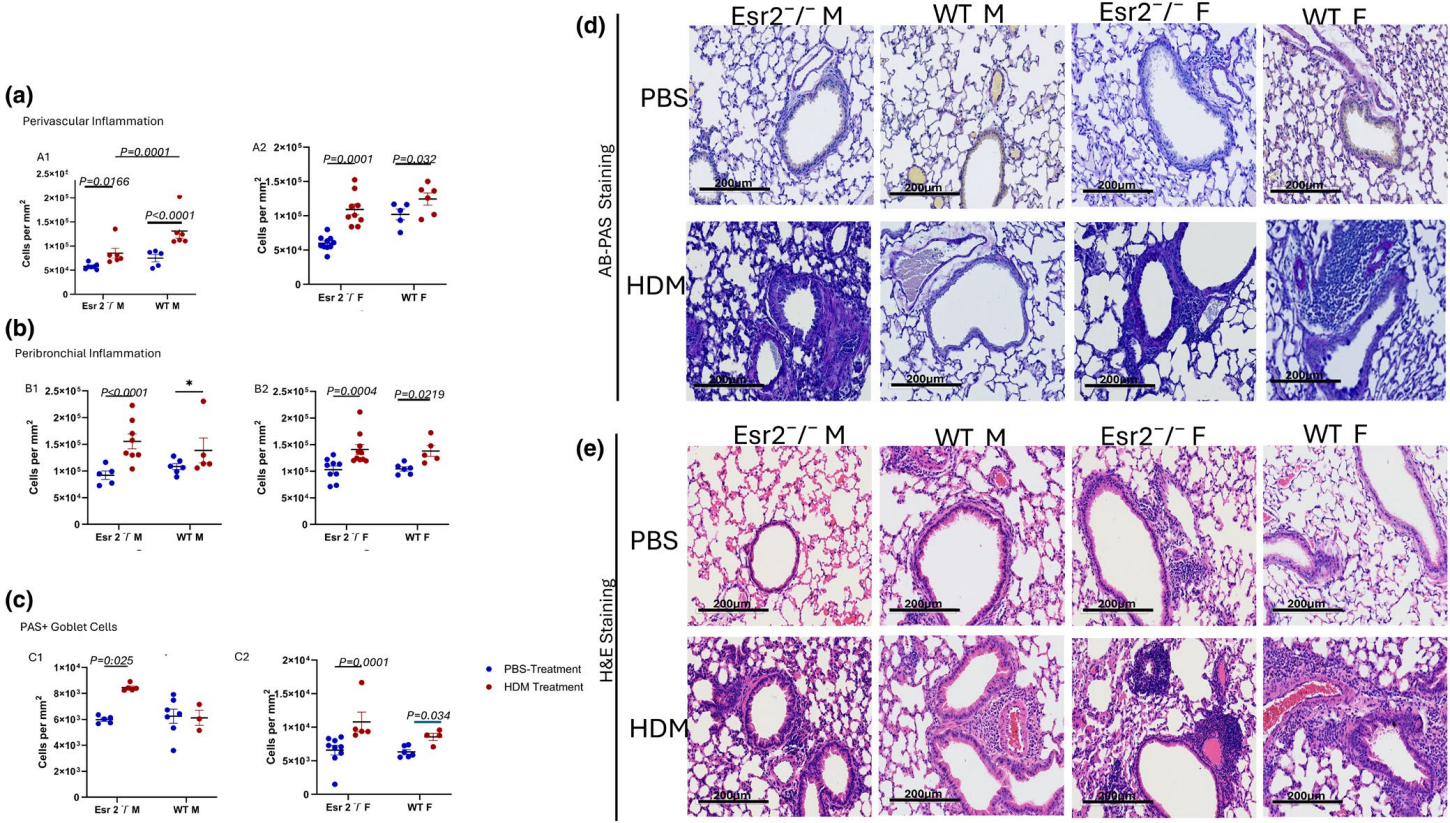
ERβ regulates compartment‐specific inflammatory remodeling and epithelial mucus metaplasia following chronic HDM exposure. (A1, A2) Quantification of perivascular inflammatory cell density in male and female WT and Esr2^−^/^−^ mice following chronic PBS or HDM exposure. (B1, B2) Quantification of peribronchial inflammatory cell infiltration demonstrating increased airway‐wall inflammation following HDM exposure. (C1–C2) Quantification of PAS+ goblet‐cell density demonstrating enhanced epithelial mucus metaplasia in HDM‐treated mice, particularly females. (D) Representative Alcian Blue–Periodic Acid–Schiff (AB‐PAS)‐stained lung sections demonstrating mucus‐producing goblet cells and airway remodeling following HDM exposure. (E) Representative hematoxylin and eosin (H&E)‐stained lung sections demonstrating perivascular and peribronchial inflammatory infiltrates. Scale bars = 200 μm. Data are presented as mean ± SEM. Exact *p*‐values are indicated on the graphs. Blue symbols represent PBS‐treated mice and red symbols represent HDM‐treated mice. HDM, house dust mite; WT, wild type.

Chronic HDM exposure elicited robust structural and inflammatory remodeling across lung compartments, with significant effects on perivascular and peribronchial inflammatory cell infiltration and epithelial goblet‐cell metaplasia (Figure 2a–e). Although genotype‐dependent differences did not always reach significance at the global model level, multiple sex‐ and genotype‐specific interactions emerged from post hoc analyses, revealing a complex ERβ‐mediated regulation of allergen‐driven tissue pathology.

#### 3.2.1 Perivascular inflammation

Perivascular inflammatory cell density was strongly influenced by HDM exposure (*p* < 0.001) and sex (*p* = 0.017), with significant genotype × sex (*p* = 0.033) and genotype × exposure × sex interactions (*p* = 0.012), indicating that ERβ‐dependent effects differ between males and females. In female Esr2^−^/^−^ mice, HDM exposure nearly doubled perivascular inflammation relative to PBS (112,447 ± 7150 vs. 57,815 ± 6740 cells/mm^2^; *p* = 0.0001), and a significant increase was also detected in male Esr2^−^/^−^ mice (85,149 ± 8260 vs. 55,927 ± 8260 cells/mm^2^; *p* = 0.0166). WT animals similarly showed HDM‐induced elevations, with WT females increasing to 130,500 ± 9050 vs. 102,072 ± 9050 cells/mm^2^ (*p* = 0.0320) and WT males demonstrating the most dramatic increase (141,612 ± 10,100 vs. 74,833 ± 9050 cells/mm^2^; *p* < 0.0001) (Figure 2A1, A2). Genotype comparisons also revealed sex‐specific effects. Among HDM‐treated males, WT mice exhibited significantly greater perivascular inflammation than Esr2^−^/^−^ males (difference = 56,464 ± 13,100 cells/mm^2^; *p* = 0.0001), while HDM‐treated female WT and Esr2^−^/^−^ mice did not differ. Under PBS, however, WT females displayed higher inflammation than Esr2^−^/^−^ females (*p* = 0.0003). When restricted to HDM‐only groups, WT males again showed greater inflammation than Esr2^−^/^−^ males (*p* = 0.0183), whereas no significant differences were seen among female genotypes (Figure 2A1, A2).

#### 3.2.2 Peribronchial inflammation

HDM exposure also significantly increased peribronchial inflammatory infiltration in all groups (*p* < 0.001), while genotype and sex did not show significant main effects. In female Esr2^−^/^−^ mice, HDM elevated peribronchial cell density to 146,227 ± 8260 vs. 103,002 ± 7780 cells/mm^2^ under PBS (difference = 43,225 ± 11,300; *p* = 0.0004). Male Esr2^−^/^−^ mice exhibited an even larger response (162,708 ± 8830 vs. 92,030 ± 10,400 cells/mm^2^; difference = 70,679 ± 13,700; *p* < 0.0001). In WT mice, females showed a significant increase (138,090 ± 10,400 vs. 104,421 ± 9530 cells/mm^2^; *p* = 0.0219), whereas WT males did not exhibit a statistically significant HDM–PBS difference (Figure 2B1, B2).

#### 3.2.3 PAS‐positive goblet‐cell hyperplasia

HDM also induced significant goblet‐cell metaplasia (exposure *p* < 0.001; sex *p* = 0.033), with genotype trending toward significance (*p* = 0.055). In female Esr2^−^/^−^ mice, HDM produced a striking increase in PAS^+^ cells (10,792 ± 743 vs. 6598 ± 554 cells/mm^2^; *p* = 0.0001). Male Esr2^−^/^−^ mice demonstrated a significant but smaller increase (8455 ± 743 vs. 5985 ± 743 cells/mm^2^; *p* = 0.0248). In WT mice, females again showed a significant elevation (8574 ± 830 vs. 6105 ± 743 cells/mm^2^; *p* = 0.0337), while WT males did not (Figure 2C1, C2). Among HDM‐treated mice, Esr2^−^/^−^ females displayed significantly higher PAS^+^ cell density than WT males (difference = 4672 ± 1420 cells/mm^2^; *p* = 0.0263), although no significant differences were detected between Esr2^−^/^−^ and WT females (Figure 2C1, C2).

### 3.3 Sex‐ and ERβ‐dependent gene expression profiles in HDM‐induced allergic airway inflammation

To determine how ERβ regulates immune, epithelial, and metabolic transcriptional programs during allergic airway inflammation, we performed whole‐lung RNA sequencing in male and female Esr2^−^/^−^ and WT mice following 5 weeks of HDM or PBS exposure. Differential expression analysis revealed extensive genotype‐ and sex‐dependent transcriptional remodeling in response to chronic allergen challenge, with distinct transcriptional signatures observed across exposure, sex, and genotype comparisons.

**FIGURE 3:**
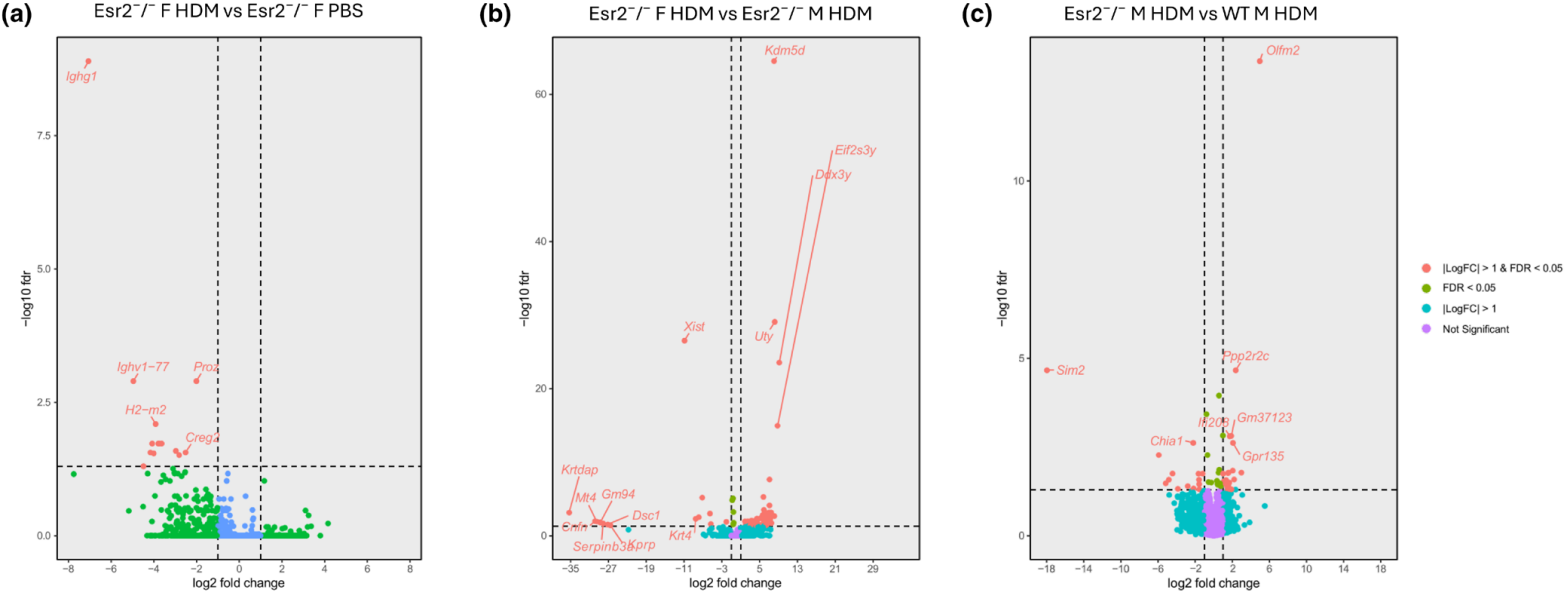
ERβ deficiency alters sex‐dependent transcriptional programs following chronic HDM exposure. (a) Volcano plot showing differential gene expression in Esr2^−^/^−^ female lungs following HDM exposure compared with PBS‐treated Esr2^−^/^−^ female controls. Chronic allergen exposure resulted in downregulation of immunoglobulin‐associated and epithelial differentiation genes, including *Ighg1*, *Ighv1‐77*, *H2‐m2*, and *Creg2*. (b) Volcano plot comparing HDM‐treated Esr2^−^/^−^ female and male lungs demonstrating pronounced sex‐dependent transcriptional differences. Female lungs exhibited altered epithelial differentiation signatures, whereas male lungs showed increased expression of Y chromosome–associated genes including *Kdm5d*, *Ddx3y*, and *Eif2s3y*. (c) Volcano plot comparing HDM‐treated Esr2^−^/^−^ and WT male lungs demonstrating ERβ‐dependent alterations in inflammatory, metabolic, and remodeling‐associated pathways. Differentially expressed genes included *Olfm2*, *Ppp2r2c*, *Ifi208*, *Gpr135*, *Sim2*, and *Chia1*. Red symbols indicate genes with |logFC| >1 and FDR <0.05; green symbols indicate genes with |logFC| >1; cyan symbols indicate genes with FDR <0.05; and purple symbols indicate genes that were not significantly differentially expressed. Highlighted genes represent selected transcripts of biological relevance, *n* = 3/group.

In Esr2^−^/^−^ females, HDM exposure resulted in widespread downregulation of genes associated with humoral immunity and epithelial differentiation. Multiple immunoglobulin heavy and variable chain genes, including *Ighg1, Ighv1‐77, Ighv1‐85, Ighv5‐15, Igkv2‐137, Igkv6‐13*, and *Igkv3‐7*, as well as complement‐ and antigen‐presentation–related genes such as *Proz* and *H2‐m2*, were significantly downregulated (Figure 3a). Additional downregulation of structural and regulatory genes, including *Creg2*, suggested attenuated epithelial integrity and reduced B‐cell activity, consistent with impaired humoral immune responses in the absence of ERβ. Comparison of HDM‐exposed Esr2^−^/^−^ females to Esr2^−^/^−^ males revealed striking sex‐linked differences. Males displayed upregulation of *Y*‐linked transcripts (*Uty, Ddx3y, Eif2s3y*, and *Kdm5d*), while females exhibited significant downregulation of epithelial keratinization and differentiation markers (*Krtdap, Mt4, Cnfn, Dsc1, Krt4, Kprp*, and *Serpinb3a*) (Figure 3b). Collectively, these results indicate that ERβ deficiency disrupts epithelial barrier and differentiation programs in female lungs.

When compared to HDM‐treated WT females, *Olfm2* was markedly upregulated in Esr2^−^/^−^ females (Figure 3c). This gene, which encodes a secreted glycoprotein implicated in extracellular matrix signaling and neuroimmune regulation, may reflect compensatory remodeling or stress adaptation in the absence of ERβ‐mediated epithelial protection. In contrast, HDM‐treated Esr2^−^/^−^ males exhibited a distinct gene expression profile dominated by cytoskeletal remodeling and metabolic pathway activation. The keratinocyte‐associated gene *Krtdap* was strongly upregulated, while several immune and structural genes, including *Dsg3, Hrnr, Ighg1, Mir6236, Ighv‐63*, and *Slc26a4*, were significantly reduced compared with PBS‐treated KO males (Figure S1A).

Relative to HDM‐treated WT males, Esr2^−^/^−^ males also displayed increased expression of *Olfm2, Ppp2r2c, Gm37123, Ifi208*, and *Gpr135*, coupled with repression of *Sim2* and *Chia1* (Figure S1B). *Ifi208*, an interferon‐inducible transcript, indicates possible shifts toward antiviral or innate immune signaling, while *Chia1* (acidic mammalian chitinase), a well‐characterized Th2 effector molecule, was notably decreased, suggesting a dampened classic Th2 inflammatory axis in the absence of ERβ. *Sim2*, a transcription factor involved in epithelial differentiation, was also suppressed, implying impaired epithelial repair capacity. Together, these changes highlight a transition toward remodeling and metabolic reprogramming in the ERβ‐deficient male lung. On the other hand, in WT females (Figure S1C), HDM exposure produced a robust allergic transcriptional signature with activation of immunoglobulin, inflammatory, and epithelial remodeling pathways. Genes encoding immunoglobulin chains (*Ighv1‐54, Igha, Igkv10‐96, Ighg2b, Igkv3‐12, Ighv5‐12, Ighe, Igkc*, and *Igkj1*) were upregulated, indicating B‐cell activation and antibody class switching. Concurrent induction of *Foxp3, Ctss, Ccl6, Cxcl9, Ccl20, Tnf, Il17a*, and *Il4i1* reflected a mixed Th2/Th17 inflammatory milieu. Increased expression of myeloid and macrophage activation markers, including *Trem2, Slamf7, Adgre1*, and *Cd68*, further indicated innate immune engagement. Epithelial differentiation markers (*Krt13, Krt4, Krt78*) and tight junction and secretory genes (*Cldn10, Scgb1a1, Scgb1c1*) were also induced, consistent with epithelial remodeling and mucus hypersecretion. Upregulation of matrix metalloproteinases (*Mmp12, Mmp13*, and *Mmp19*) and chitinase‐like genes (*Chia1, Chil3*, and *Chil4*) pointed to extensive extracellular matrix turnover and tissue remodeling. At the same time, increased expression of serine protease inhibitors (*Serpinb3a, Serpina10*, and *Serpina3* family members) suggested activation of counter‐regulatory mechanisms that limit protease‐mediated damage.

WT males also exhibited gene expression changes indicative of epithelial and structural stress in response to HDM exposure. Compared with PBS controls, *Dsg3, Ighg1, Dsg1b, Hrnr*, and *Igha* were downregulated, while *Sim2, Krtdap*, and *Cnfn* were upregulated, reflecting epithelial turnover and differentiation during ongoing tissue injury (Figure S1D). In Esr2^−^/^−^ males (relative to WT males), we observed repression of genes involved in lipid metabolism and oxidative stress responses (*Bche, Thrsp, Smoc1, Dgat2, Agpat2, Mir6236, Copg1, Naxd, Lep, Pcx, Acly, Mrap, Nnt*, and *Sim2*) and induction of *Olfm2, Ifi208, Ppp2r2c, Casp8*, and *Nkx2‐1*. These patterns imply ERβ‐dependent regulation of mitochondrial and metabolic balance, with upregulation of *Casp8* and *Nkx2‐1*.

**FIGURE 4:**
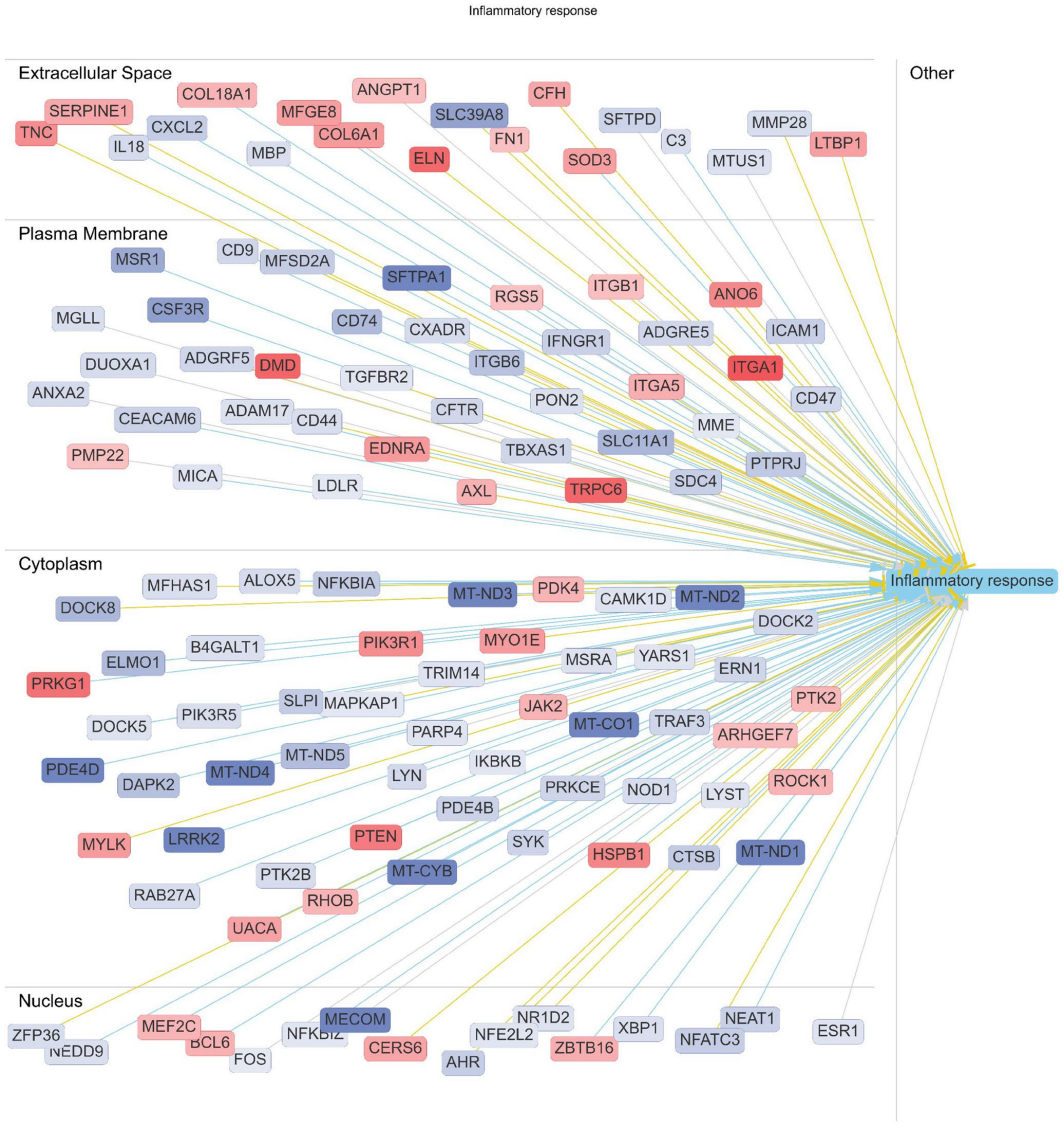
ERβ‐dependent inflammatory response network in male lungs following chronic HDM exposure. Ingenuity Pathway Analysis (IPA) network demonstrating ERβ‐dependent transcriptional regulation of inflammatory and immunometabolic pathways in HDM‐treated male lungs. Differentially expressed genes associated with extracellular matrix remodeling, inflammatory signaling, oxidative stress, mitochondrial metabolism, and membrane receptor activity are organized according to cellular compartment, including extracellular space, plasma membrane, cytoplasm, and nucleus. Network analysis identified coordinated regulation of genes linked to inflammatory response pathways, including integrin signaling, cytokine signaling, mitochondrial stress responses, and cytoskeletal remodeling. Node color represents relative gene expression changes. Red nodes = increased expression; blue nodes = decreased expression in Esr2^−^/^−^ male lungs relative to WT male lungs following HDM exposure. Yellow lines indicate predicted activation, blue lines indicate predicted inhibition, and gray lines indicate indirect or unspecified interactions. Genes are grouped by cellular compartment.

In males, comparison of Esr2^−^/^−^ and WT lungs (Figure 4) showed that ERβ loss reshaped inflammatory signaling across multiple cellular compartments rather than inducing broad inflammatory activation. Network analysis identified altered expression of genes involved in cell adhesion, extracellular matrix organization, and chemokine signaling, alongside engagement of cytoplasmic kinase cascades, NF‐κB regulatory nodes, and calcium/contractile machinery. Prominent representation of mitochondrial and metabolic genes indicated altered immunometabolic coupling, suggesting that ERβ loss in males reprograms inflammatory tone and spatial organization rather than driving epithelial remodeling or severe mechanical dysfunction.

**FIGURE 5:**
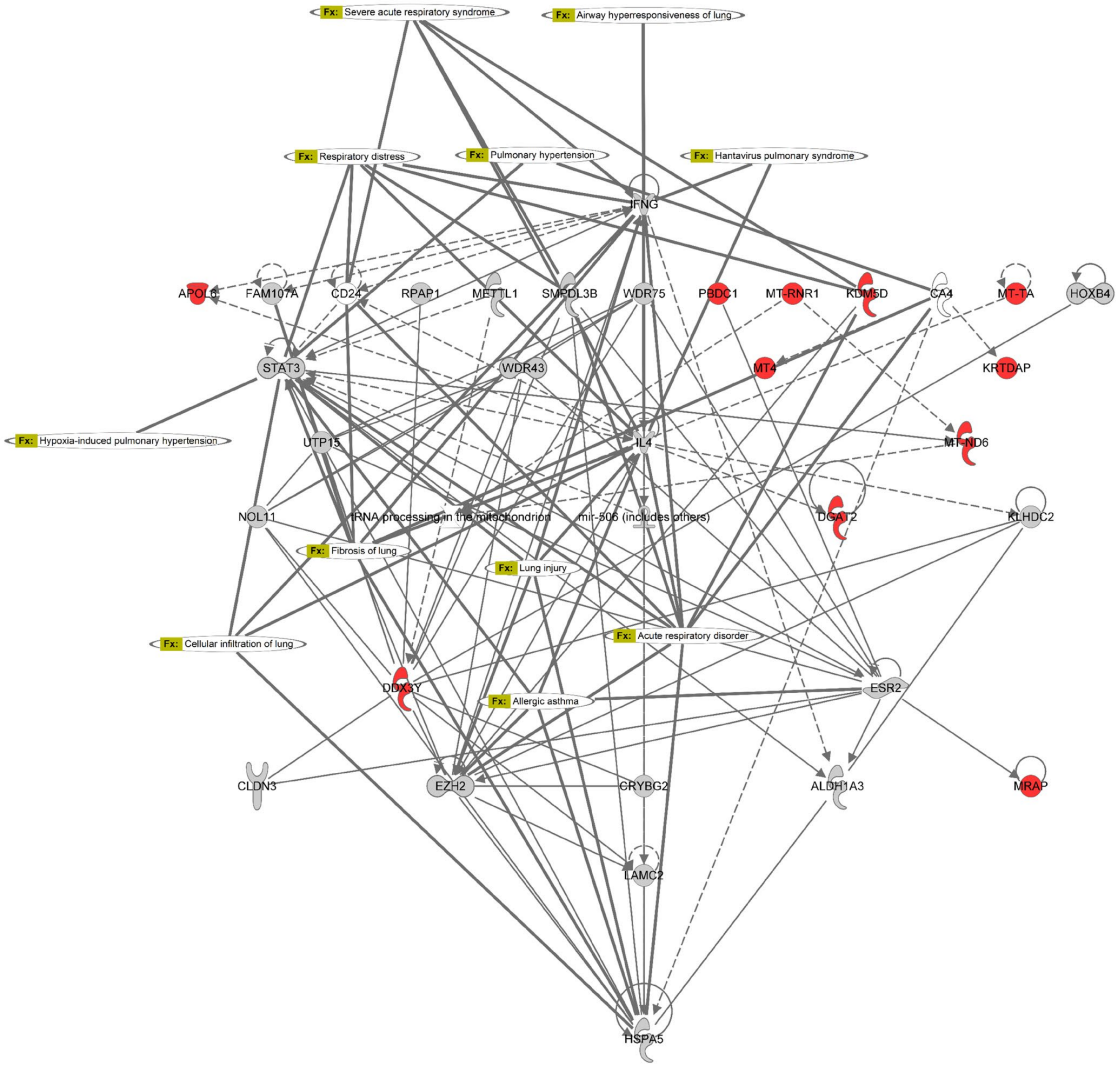
ERβ‐associated remodeling and airway injury network following chronic HDM exposure. Ingenuity Pathway Analysis (IPA) network demonstrating ERβ‐associated molecular interactions linked to airway remodeling, respiratory stress, epithelial dysfunction, and inflammatory lung injury following chronic HDM exposure. The network highlights coordinated interactions among mitochondrial genes, stress‐response mediators, extracellular matrix regulators, and inflammatory signaling pathways associated with severe allergic airway disease, pulmonary hypertension, airway hyperresponsiveness, and tissue remodeling. Differentially expressed genes associated with epithelial remodeling and inflammatory signaling, including *KRTDAP*, mitochondrial transcripts, and stress‐response regulators, are integrated into a predicted interaction network centered on ERβ‐dependent airway injury pathways. Node color represents relative gene expression changes. Red nodes = increased expression in Esr2^−^/^−^ female lungs relative to Esr2^−^/^−^ male lungs following HDM exposure. Yellow labels indicate predicted disease functions or biological processes. Solid lines represent direct interactions, and dashed lines represent indirect or predicted interactions identified by IPA.

**FIGURE 6:**
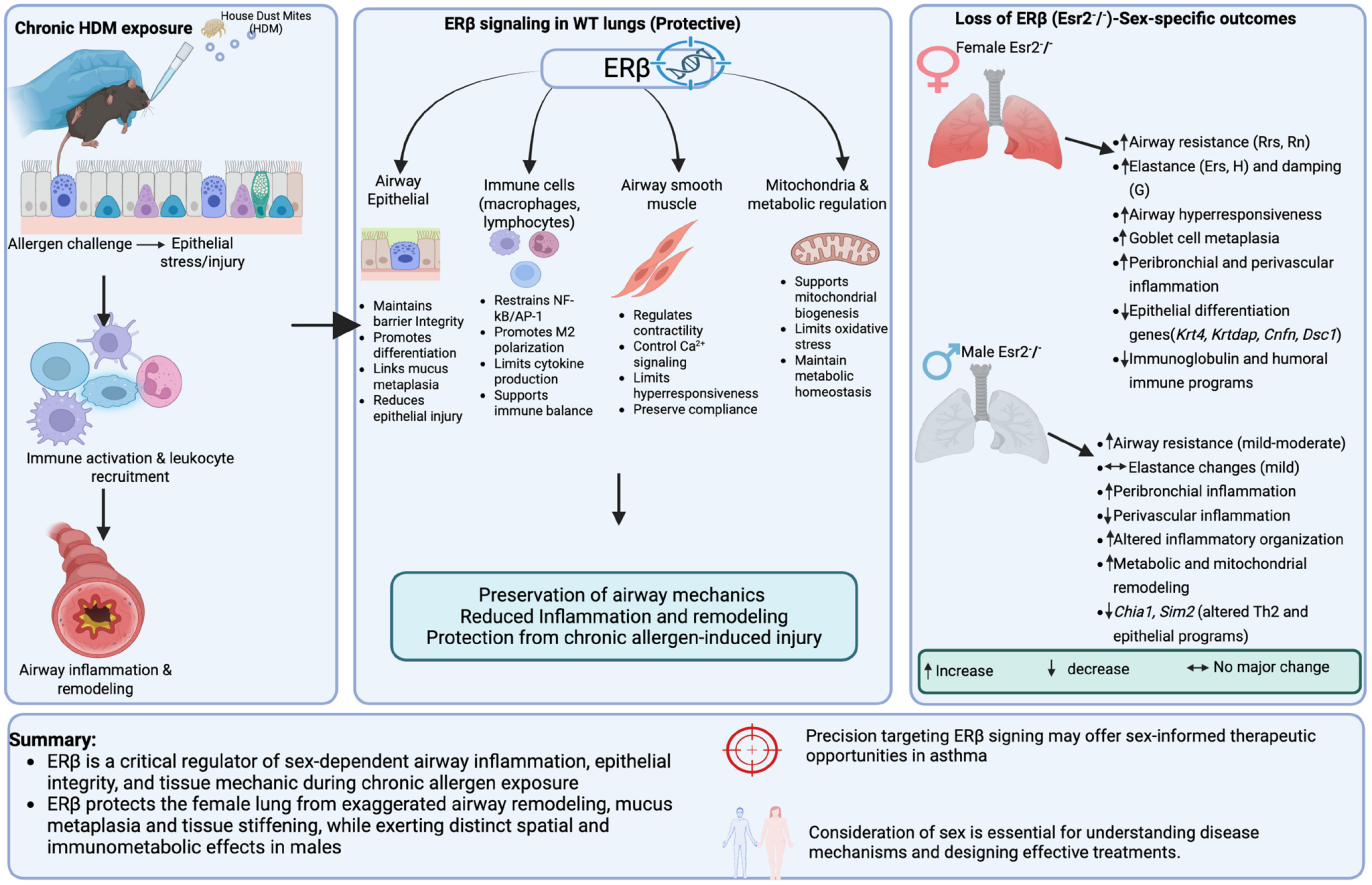
Proposed model of ERβ‐dependent regulation of sex‐specific airway remodeling and mechanical dysfunction following chronic HDM exposure. Chronic house dust mite (HDM) exposure induces airway inflammation, epithelial injury, leukocyte recruitment, and structural remodeling in both male and female lungs. In WT mice, ERβ signaling maintains airway epithelial integrity, regulates immune activation, preserves airway smooth muscle homeostasis, and supports mitochondrial and metabolic function, thereby limiting airway hyperresponsiveness, mucus metaplasia, and tissue injury. Loss of ERβ produces sex‐dependent pathological outcomes. Female Esr2^−^/^−^ mice develop severe airway remodeling characterized by increased airway resistance, tissue elastance, tissue damping, mucus metaplasia, and peribronchial/perivascular inflammation, accompanied by suppression of epithelial differentiation and humoral immune programs. In contrast, male Esr2^−^/^−^ mice exhibit altered inflammatory organization, metabolic and mitochondrial remodeling, and comparatively milder mechanical dysfunction, including reduced perivascular inflammation relative to WT males. Together, these findings identify ERβ as a critical regulator of sex‐dependent airway inflammation, epithelial integrity, and tissue mechanics during chronic allergen exposure and support ERβ signaling as a potential target for sex‐informed therapeutic strategies in asthma. Ers, respiratory system elastance; ERβ, estrogen receptor beta; G, tissue damping; H, tissue elastance; HDM, house dust mite; Rn, Newtonian resistance; Rrs, total respiratory resistance; WT, wild type.

In contrast, direct comparison of Esr2^−^/^−^ females and Esr2^−^/^−^ males (Figure 5) revealed a fundamentally different transcriptional architecture. Esr2^−^/^−^ females exhibited coordinated activation of gene networks governing epithelial differentiation, extracellular matrix remodeling, immune activation, and airway surface regulation, consistent with their pronounced airway hyperresponsiveness, tissue stiffening, goblet‐cell metaplasia, and airway‐wall inflammation. Network connectivity in females spanned extracellular, membrane, cytoplasmic, and nuclear compartments, indicating synchronized engagement of immune–epithelial remodeling programs. A summary schematic integrating the principal physiological, histopathological, and transcriptomic findings and illustrating the proposed ERβ‐dependent mechanisms underlying sex‐specific airway remodeling and mechanical dysfunction following chronic HDM exposure is shown in Figure 6. A complementary overview of the major physiological, inflammatory, and transcriptomic differences across sex, genotype, and exposure comparisons is provided in Table 1.

**TABLE 1:** Summary of sex‐, genotype‐, and HDM‐dependent physiological, inflammatory, and transcriptomic responses.

Comparison	Lung mechanics	Histopathology	Transcriptomic changes	Biological interpretation
Esr2^−^/^−^ female HDM vs. PBS	Marked increases in Rrs, Ers, Rn, G, and H	Increased perivascular and peribronchial inflammation; severe PAS^+^ goblet‐cell hyperplasia	Downregulation of immunoglobulin and epithelial differentiation genes (*Ighg1*, *Ighv1‐77*, *Krt4*, *Dsc1*)	ERβ loss promoted severe airway remodeling and mechanical dysfunction in females
Esr2^−^/^−^ male HDM vs. PBS	Increased Rrs, Rn, G, and H, but less severe than females	Increased peribronchial inflammation and moderate goblet‐cell hyperplasia	Altered remodeling and metabolic genes (*Krtdap*, *Dsg3*, *Slc26a4*)	ERβ deficiency altered inflammatory organization and remodeling in males
WT female HDM vs. PBS	Mild increases in airway resistance with preserved distal mechanics	Increased inflammation and mucus metaplasia	Upregulation of immunoglobulin, inflammatory, and epithelial remodeling genes (*Ighg1*, *Igha*, *Mmp12*, *Serpinb3a*)	WT females mounted coordinated immune and epithelial repair responses
WT male HDM vs. PBS	Modest airway mechanical changes	Increased perivascular inflammation with limited goblet‐cell hyperplasia	Differential expression of epithelial remodeling genes (*Dsg3*, *Krtdap*, *Sim2*)	WT males exhibited milder remodeling and inflammatory responses
Esr2^−^/^−^ female vs. male HDM	Females exhibit greater tissue stiffness and airway dysfunction	Females show enhanced mucus metaplasia and inflammatory remodeling	Female‐specific suppression of epithelial differentiation genes and altered sex chromosome‐associated transcripts	ERβ deficiency unmasked female‐biased susceptibility to severe allergic airway disease
Esr2^−^/^−^ male vs. WT male HDM	Altered inflammatory distribution with moderate mechanical dysfunction	Increased airway‐wall inflammation	Upregulation of *Olfm2*, *Ifi208*, *Ppp2r2c*; downregulation of *Chia1* and *Sim2*	ERβ regulated inflammatory and metabolic signaling in male lungs

## 4 DISCUSSION

The present study demonstrates that ERβ represents a major sex‐dependent regulator of airway mechanics, inflammatory architecture, gene expression, and epithelial remodeling in chronic allergic airway disease. By integrating lung mechanics, histopathology, and transcriptomics, our study shows that loss of ERβ unmasks a striking female‐specific susceptibility to allergen‐induced airway dysfunction. This finding provides a mechanistic framework for the persistent global observation that women bear a disproportionate burden of severe asthma and airway remodeling in adulthood, despite comparable allergen exposure and treatment access (Cao et al., 2025; Ekpruke & Silveyra, 2022; Pinkerton et al., 2015; Silveyra et al., 2026; Yung et al., 2018).

Across all physiological readouts, female Esr2^−^/^−^ mice exhibited the most severe impairment, including exaggerated increases in Rrs, Rn, Ers, G and H. These coordinated changes indicate both enhanced airway narrowing and profound alterations in distal lung mechanics, consistent with increased parenchymal stiffness, tissue heterogeneity, and small‐airway closure. Such a mechanical phenotype is characteristic of severe, remodeling‐dominant asthma, which is known to be overrepresented in women and poorly responsive to conventional bronchodilator therapy (Barnes, 2008; Ekpruke & Silveyra, 2022; Pinkerton et al., 2015). Importantly, WT females were relatively protected from these mechanical consequences despite robust inflammatory responses, identifying ERβ as a critical protective determinant of lung functional integrity in females.

Histological analyses revealed that ERβ regulates inflammatory architecture in a compartment‐ and sex‐specific manner. In the perivascular compartment, HDM induced substantial inflammation across all groups; however, ERβ loss amplified the HDM response in females relative to PBS, whereas WT males displayed the highest absolute perivascular inflammation following allergen exposure. In contrast, peribronchial inflammation was selectively amplified by ERβ deficiency in males, while females showed strong HDM responses independent of genotype. These divergent patterns suggest that ERβ does not act as a global suppressor of inflammation but rather functions as a context‐dependent regulator whose effects vary by sex, vascular versus airway‐wall niche, and inflammatory microenvironment. Such compartmental specificity is consistent with evidence that estrogen receptors modulate endothelial activation, leukocyte trafficking, and macrophage polarization through distinct downstream pathways depending on cell type and hormonal context (Hou & Adzika, 2025; Keselman & Heller, 2015; Kovats, 2015).

The epithelial compartment emerged as a key site of ERβ‐dependent protection, particularly in females. Female Esr2^−^/^−^ mice exhibited the greatest PAS^+^ goblet‐cell hyperplasia, aligning closely with their exaggerated tissue mechanical dysfunction. This relationship supports a model in which ERβ maintains epithelial differentiation and suppresses pathological mucus metaplasia during chronic allergen exposure. Transcriptomic analysis reinforced this interpretation, revealing suppression of epithelial differentiation markers and immunoglobulin‐related genes in Esr2^−^/^−^ females, indicative of impaired epithelial‐immune coordination. Prior studies have shown that ERβ regulates epithelial homeostasis, mitochondrial function, and oxidative stress responses (Chen et al., 2022; Chen, Chen, et al., 2007; Chen, Russo, et al., 2007; Gong et al., 2014; Novelle et al., 2023; Pedram et al., 2010; Rius‐Pérez et al., 2020), all of which are critical for maintaining airway barrier integrity under inflammatory stress.

Sex differences in immune regulation further contextualize these findings. Estrogen signaling is known to shape innate and adaptive immune responses, including macrophage polarization, cytokine production, and Th2/Th17 balance (Keselman et al., 2017; Kovats, 2015). Our data suggest that ERβ acts as a buffering receptor in females, restraining excessive immune–epithelial crosstalk that would otherwise drive mucus hypersecretion and tissue stiffening. In males, however, ERβ appears to modulate inflammatory distribution rather than magnitude, dampening peribronchial inflammation while permitting greater vascular involvement. These divergent outcomes underscore the importance of sex‐specific nuclear receptor signaling networks, which may be influenced by epigenetic regulation and chromatin context (Song et al., 2022; Weiser et al., 2009). Although this study was performed in adult mice under a defined hormonal state, the findings suggest that female vulnerability to ERβ loss may be greatest during periods of endocrine fluctuation, when estrogen availability and receptor signaling are changing. Such windows may include puberty, cyclic hormonal variation, pregnancy and the postpartum period, and the transition to perimenopause or menopause. During these stages, reduced ERβ signaling may be less able to preserve epithelial barrier integrity and restrain inflammatory remodeling, thereby amplifying susceptibility to allergen‐induced airway dysfunction. Thus, the impact of ERβ deficiency likely depends not only on sex, but also on developmental stage and the estrogen receptor–ligand environment.

The integration of transcriptomic and physiological data supports ERβ as a molecular hub linking hormone signaling to mechanical and inflammatory outcomes. Disruption of ERβ signaling altered immune gene networks, epithelial differentiation programs, and metabolic pathways associated with mitochondrial function and oxidative stress. Such pathways are increasingly recognized as central drivers of airway remodeling and steroid‐resistant asthma (Barnes, 2008; Chen et al., 2024; Rius‐Pérez et al., 2020). Notably, ERβ‐dependent regulation of metabolic and redox pathways may influence fibroblast activation, extracellular matrix turnover, and smooth muscle behavior, providing a mechanistic bridge between inflammation and tissue mechanics.

Sex‐based comparisons across genotypes reinforced the role of both chromosomal and hormonal factors in shaping airway transcription. Male‐biased genes included the *Y*‐linked *Kdm5d, Uty, Ddx3y*, and *Eif2s3y* as well as the developmental regulators *Sim2, Taf15*, and *Nkx2‐1*, whereas females expressed higher levels of *Xist, Kdm6a, Kdm5c, Ddx3x*, and epithelial differentiation genes such as *Krt13, Krt4, Krtdap*, and *Cnfn*. The epithelial‐derived cytokine *Il36g* was preferentially expressed in females, suggesting enhanced epithelial inflammatory responses. Female‐biased upregulation of *H19*, an estrogen‐responsive long noncoding RNA implicated in fibrosis and airway remodeling, further highlighted sex‐specific regulation of tissue remodeling processes. Integration of these two network analyses provides critical mechanistic insight into how ERβ governs sex‐biased susceptibility to allergic airway disease. In males, ERβ loss does not precipitate the severe airway remodeling or mechanical impairment observed in females. Instead, the Esr2^−^/^−^ male network reveals a redistribution of inflammatory signaling, characterized by altered cell–matrix interactions, vascular–immune communication, stress‐response pathways, and mitochondrial metabolism. This transcriptional profile aligns with the relatively modest mechanical abnormalities and compartment‐specific inflammatory changes observed in Esr2^−^/^−^ males, indicating that ERβ in the male lung primarily regulates the organization and metabolic integration of inflammation, rather than its magnitude.

By contrast, the Esr2^−^/^−^ female network demonstrates that ERβ loss removes a critical restraint on epithelial–immune coupling and tissue remodeling. In females, ERβ deficiency permits synchronized activation of inflammatory, epithelial differentiation, and extracellular matrix programs, producing widespread airway‐wall inflammation, mucus hypersecretion, distal tissue stiffening, and severe airway hyperresponsiveness. The contrast between the two networks indicates that ERβ acts as a sex‐specific key modulator: in females, it suppresses remodeling‐dominant inflammatory cascades, whereas in males it constrains inflammatory localization and metabolic stress responses.

Importantly, the direct Esr2^−^/^−^ female–male comparison demonstrates that these differences persist even in the complete absence of ERβ, indicating that sex‐dependent regulatory programs are intrinsic to the lung and become unmasked when ERβ signaling is lost. Compensatory hormone‐responsive pathways evident in Esr2^−^/^−^ males may further buffer against epithelial dysfunction, explaining why ERβ loss does not phenocopy the severe female phenotype. Together, these findings provide a unified mechanistic framework linking ERβ signaling to sex‐biased airway mechanics, inflammatory architecture, and epithelial remodeling, and they identify ERβ as a central determinant of why allergic airway disease progresses toward a more severe, remodeling‐dominant phenotype in females.

Clinically, these findings have important implications. Current asthma therapies largely ignore sex as a biological variable, despite well‐documented differences in disease severity, progression, and treatment response (Ekpruke & Silveyra, 2022; Pinkerton et al., 2015; Silveyra et al., 2026; Yung et al., 2018). Our data suggest that ERβ activation may be particularly beneficial in females with remodeling‐dominant, mucus‐rich asthma phenotypes, whereas indiscriminate modulation of estrogen signaling could yield divergent effects in males. This aligns with emerging evidence that estrogen receptors, including ERβ and GPER, exert nonredundant and sometimes opposing roles in chronic inflammatory diseases (Arterburn & Prossnitz, 2023; Chen, Russo, et al., 2007; Itoga et al., 2024; Na et al., 2018; Prossnitz & Barton, 2011). Identification of ERβ‐regulated transcriptional signatures may therefore enable precision stratification of patients most likely to benefit from hormone‐informed or adjunctive biologic therapies (Carriera et al., 2023; Liu et al., 2025). The integrated model presented in Figure 6 summarizes the proposed mechanistic framework emerging from this study, whereby ERβ signaling coordinates epithelial integrity, immune regulation, mitochondrial homeostasis, and airway mechanics in a sex‐dependent manner during chronic allergen exposure. Loss of ERβ disproportionately amplifies inflammatory remodeling and tissue dysfunction in females, while altering inflammatory organization and metabolic signaling in males.

Several limitations warrant consideration. The use of constitutive Esr2 knockout mice does not distinguish between developmental and adult‐onset roles of ERβ; therefore, conditional and cell‐type–specific deletions will be essential to define epithelial, endothelial, immune, and smooth muscle contributions to allergic airway disease. In addition, the present study was performed in adult mice at a single hormonal stage and did not account for estrous cycle variation or changes in estrogen availability across the lifespan. Future studies examining puberty, pregnancy, reproductive aging, and hormone manipulation will be important to determine how fluctuations in estrogen signaling influence ERβ‐dependent airway responses. Although the HDM model captures many hallmark features of chronic allergic asthma, it does not encompass other clinically relevant endotypes such as neutrophilic or obesity‐associated asthma. Furthermore, whole‐lung transcriptomics limits cell‐specific resolution, and future studies employing single‐cell or spatial transcriptomic approaches will refine mechanistic understanding. Future investigations will also determine whether transcriptional alterations in immunoglobulin‐related genes translate into altered antibody production or shifts in B‐cell populations. Finally, although methacholine responsiveness is widely used to model airway hyperresponsiveness, translation of murine airway physiology to human disease will require validation in clinical cohorts.

In conclusion, this study establishes ERβ as a central regulator of sex‐biased allergic airway disease, coordinating airway mechanics, inflammatory architecture, epithelial remodeling, and transcriptional homeostasis. Loss of ERβ is associated with a pronounced female vulnerability to severe allergen‐induced lung dysfunction, offering a mechanistic explanation for sex differences observed in asthma epidemiology and outcomes. These findings argue strongly for the incorporation of sex and hormone receptor biology into asthma research and therapeutic design, and position ERβ as a promising target for precision intervention in chronic airway disease.

## AUTHOR CONTRIBUTIONS

**Carolyn Damilola Ekpruke:** Conceptualization; formal analysis; investigation; methodology. **Dustin Rousselle:** Investigation; methodology. **Maksat Babayev:** Investigation; methodology. **Omar Borges‐Sosa:** Investigation; methodology. **Rachel Alford:** Investigation; methodology. **Lyidia Dinwiddie:** Methodology. **David Michael Merritt:** Methodology. **Christopher Michael Hemmerich:** Methodology. **Douglas B. Rusch:** Methodology. **Sarah Bradley:** Methodology. **Matthew Louis Retzner:** Methodology. **Erik Parker:** Methodology; visualization. **Patricia Silveyra:** Conceptualization; formal analysis; investigation; methodology; project administration; resources; supervision.

## FUNDING INFORMATION

This study was supported by NIH/NHLBI, Grant/Award Number: R01HL159764 and the Indiana Clinical and Translational Sciences Institute which is funded in part by Award Number UM1TR004402 from the National Institutes of Health, National Center for Advancing Translational Sciences, Clinical and Translational Sciences Award.

## DISCLAIMERS

The content is solely the responsibility of the authors and does not necessarily represent the official views of the National Institutes of Health.

## ETHICS STATEMENT

All experimental procedures involving vertebrate animals in this study were reviewed and approved by the Institutional Animal Care and Use Committee (IACUC) of Indiana University Bloomington under protocol numbers 21‐012 and 24‐020. All research was performed in compliance with applicable federal, state, and institutional guidelines governing animal welfare and 24‐020. Every effort was made to minimize animal suffering and to reduce the number of animals used.

## Supporting information


**Figure S1.** Additional transcriptomic comparisons associated with ERβ deficiency and chronic HDM exposure. (A) Volcano plot comparing HDM‐treated Esr2^−^/^−^ and WT female lungs. *Olfm2* was identified as the primary significantly upregulated transcript in Esr2^−^/^−^ female lungs relative to WT controls. (B) Volcano plot showing differential gene expression in Esr2^−^/^−^ male lungs following HDM exposure compared with PBS‐treated Esr2^−^/^−^ male controls. Differentially expressed genes included *Dsg3*, *Hrnr*, *Ighg1*, *Ighv‐63*, *Slc26a4*, and *Krtdap*. (C) Volcano plot comparing WT female lungs following HDM exposure versus PBS controls demonstrating activation of humoral immune and epithelial remodeling pathways, including increased expression of *Ighg1*, *Ighv1‐54*, *Igha*, *Krtdap*, and *Serpinb3a*. (D) Volcano plot comparing WT male lungs following HDM exposure versus PBS controls demonstrating altered epithelial differentiation and remodeling‐associated transcriptional programs, including differential expression of *Dsg3*, *Ighg1*, *Sim2*, *Krtdap*, and *Cnfn*. Highlighted genes represent selected transcripts of biological relevance. Red symbols indicate genes with |logFC| >1 and FDR <0.05; green symbols indicate genes with |logFC| >1; cyan symbols indicate genes with FDR <0.05; and purple symbols indicate genes that were not significantly differentially expressed.

## Data Availability

The datasets generated and analyzed during the current study have been deposited in the NCBI Gene Expression Omnibus (GEO) repository under accession number GSE317602.
