# Supplementary figures and images for: Estrogen receptor β regulates sex‐dependent airway mechanics and inflammation in a murine model of allergen exposure
Source: Physiol Rep. 2026 May 31;14(11):e70948. doi: 10.14814/phy2.70948 (PMC13240281; doi:10.14814/phy2.70948)

**A**  $Esr2^{-/-}$  F HDM vs WT F HDM

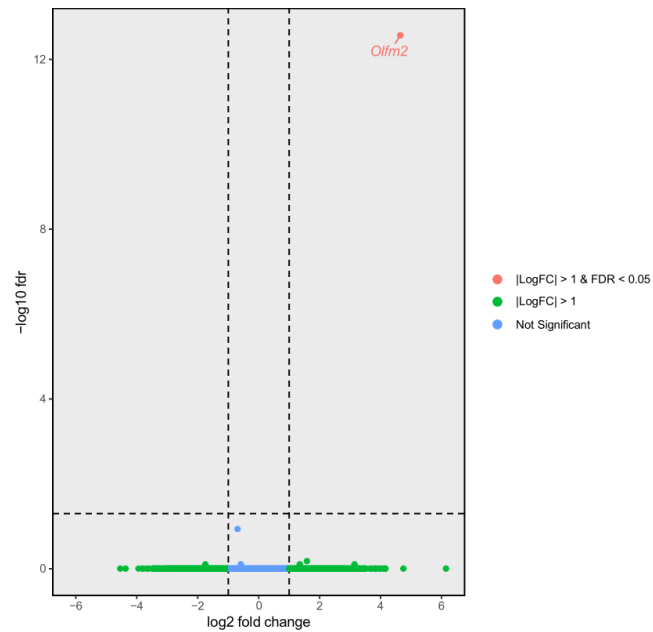

**B**  $Esr2^{-/-}$  M HDM vs  $Esr2^{-/-}$  M PBS

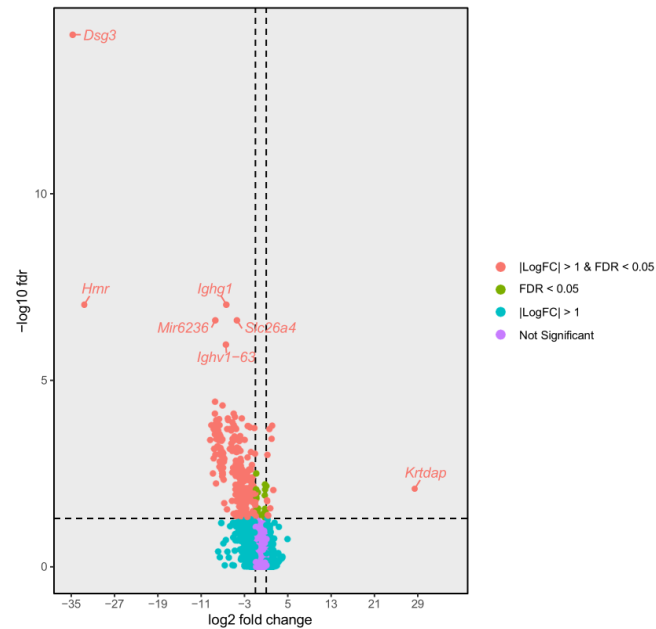

**C** WT F HDM vs WT F PBS

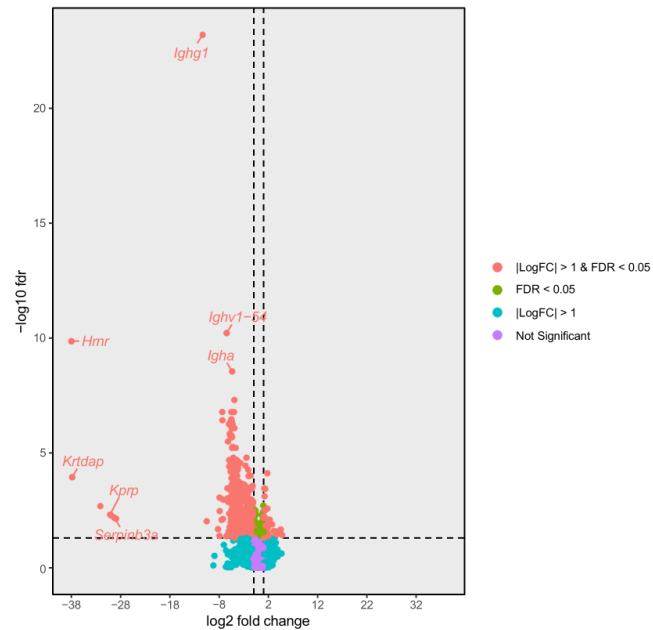

**D** WT M HDM vs WT M PBS

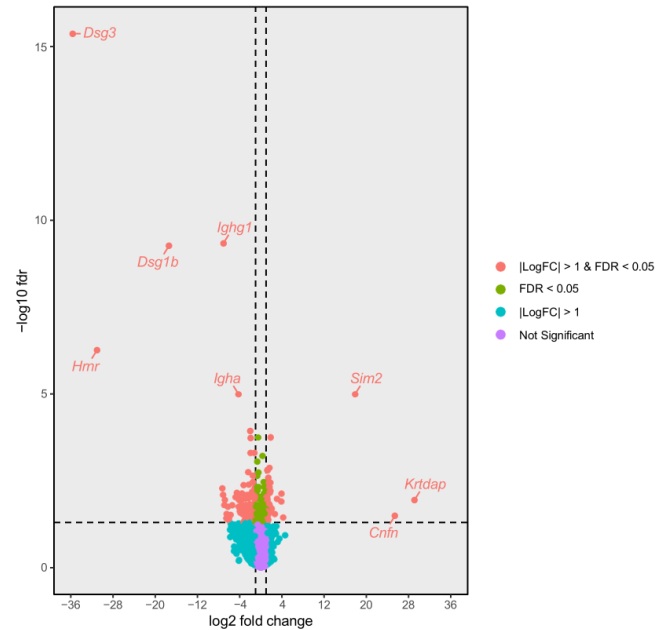

Supplement: Supplementary file 1 — Figure S1. Additional transcriptomic comparisons associated with ERβ deficiency and chronic HDM exposure. (A) Volcano plot comparing HDM‐treated Esr2−/− and WT female lungs. Olfm2 was identified as the primary significantly upregulated transcript in Esr2−/− female lungs relative to WT controls. (B) Volcano plot showing differential gene expression in Esr2−/− male lungs following HDM exposure compared with PBS‐treated Esr2−/− male controls. Differentially expressed genes included Dsg3, Hrnr, Ighg1, Ighv‐63, Slc26a4, and Krtdap. (C) Volcano plot comparing WT female lungs following HDM exposure versus PBS controls demonstrating activation of humoral immune and epithelial remodeling pathways, including increased expression of Ighg1, Ighv1‐54, Igha, Krtdap, and Serpinb3a. (D) Volcano plot comparing WT male lungs following HDM exposure versus PBS controls demonstrating altered epithelial differentiation and remodeling‐associated transcriptional programs, including differential expression of Dsg3, Ighg1, Sim2, Krtdap, and Cnfn. Highlighted genes represent selected transcripts of biological relevance. Red symbols indicate genes with |logFC| >1 and FDR <0.05; green symbols indicate genes with |logFC| >1; cyan symbols indicate genes with FDR <0.05; and purple symbols indicate genes that were not significantly differentially expressed. [file PHY2-14-e70948-s001.pdf]
